# Trends in ectopic pregnancy, hydatidiform mole and miscarriage in the largest obstetrics and gynaecology hospital in China from 2003 to 2013

**DOI:** 10.1186/s12978-016-0181-5

**Published:** 2016-05-20

**Authors:** Xue-Lian Li, Dan-Feng Du, Shang-Jie Chen, Sai-Hua Zheng, Arier C Lee, Qi Chen

**Affiliations:** Department of Gynaecology, The Hospital of Obstetrics & Gynaecology, Fudan University, 419 Fangxie Road, Shanghai, China; Shanghai Key Laboratory of Female Reproductive Endocrine Related Diseases, Shanghai, 419 Fangxie Road, Shanghai, China; Section of Epidemiology & Biostatistics, School of Population Health, The University of Auckland, Auckland, New Zealand; Department of Obstetrics & Gynaecology, The University of Auckland, New Zealand. 85 Park Road, Grafton, Auckland New Zealand

**Keywords:** Ectopic pregnancy, Hydatidiform mole, Miscarriage, Maternal age, China

## Abstract

**Background:**

Ectopic pregnancies, miscarriages and hydatidiform moles are the major types of pathological pregnancies in the early gestations of pregnancy and constitute an important public health problem. The trends and incidences of these pathological pregnancies may vary by ethnicity and geographical regions. This has not been fully investigated in the Chinese population. In this study we retrospectively report the trends of pathological pregnancies in Chinese population.

**Methods:**

Data on 22,511 women with ectopic pregnancy, hydatidiform mole and miscarriage were collected from the largest obstetrics and gynaecology hospital in China from 2003 to 2013. Data included age at diagnosis and the annual number of women with diagnosed ectopic pregnancy, hydatidiform mole and miscarriage.

**Results:**

The total number of ectopic pregnancy, hydatidiform mole and miscarriage was increased 3.5folds in 2013 compared to 2003. Ectopic pregnancy is the leading pathological pregnancy and miscarriage is increasing at a greater rate among the pathological pregnancies. The median age of women with hydatidiform mole at diagnosis significantly increased from 25.5 years to 29 years (*p* = 0.002), however the median age for other pathological pregnancies was not different between 2003 and 2013. The number of women with hydatidiform mole at diagnosis who were over 40 years old has increased. The mean maternal age is increased from 28.1 years old in 2003 to 29.4 years old in 2013 in this hospital.

**Conclusion:**

We speculate that the increased maternal age may contribute to the increase in these pathological pregnancies between 2003 and 2013 in China.

## Background

Ectopic pregnancies, miscarriages and hydatidiform moles are the most common types of pathological pregnancies in the early stage of pregnancy and are an important public health problem. Most cases of these pathological pregnancies occur in the first trimester and have some similar clinical symptoms and risk factors [1].

An ectopic pregnancy occurs when a fertilized egg implants outside the endometrial cavity, usually in the fallopian tube and it increases the risk of future infertility. Ectopic pregnancy has been reported to affect 2 % of all identified pregnancies in the United States [2, 3], and is a significant cause of morbidity and mortality in the first trimester of pregnancy. Miscarriage, a spontaneous pregnancy loss before 20 weeks of gestation, is the most common complication of early pregnancy and most miscarriages (85 %) occurs in the first trimester of pregnancy, between the 7th and 12th weeks of pregnancy. The estimated rate of miscarriage is approximate 10 to 20 % worldwide. One study has suggested that ectopic pregnancy is positively associated with miscarriage [4]. Hydatidiform mole is a growing mass of tissue in uterus that will not develop into a fetus and occurs as a result of abnormal conception. Hydatidiform mole is one of the most common complications of gestational trophoblastic diseases (GTD), which has a risk of undergoing malignant transformation and developing very early onset preeclampsia, the commonest complication of pregnancy [5]. Hydatidiform mole is rare but affects 0.6–1.1 women in 1000 healthy pregnancies [6].

The trends and incidence of ectopic pregnancy, miscarriage and hydatidiform mole may vary by the ethnicity of the population and the geographical regions. This is because ethnicity and geographical regions may be a surrogate for many potential influences on population health including access to medical care, lifestyle as well as a reflection of genetic variation within the population. Although the incidence of ectopic pregnancy has not changed substantially in the United States since the early 1990s [7, 8], other studies showed that the incidence of ectopic pregnancy has increased during the 1980s and 1990s in developed countries including the United Kingdom [4, 9, 10]. The incidence of hydatidiform mole is lower in the United States (1 in 1000 pregnancy), but it is much higher in Asia, up to 1 in 100 pregnancy in Indonesia as well Pakistan [11, 12]. One study has also indicated that incidence of hydatidiform mole is increased from 1 in 611 viable conceptions in 1997 to 1 in 528 viable conceptions in 2008 in United Kingdom [13]. These studies suggest that there are marked differences in the demographics of ectopic pregnancy, hydatidiform mole and miscarriage between developed and developing countries.

The incidence and the details of these major pathological pregnancies worldwide are dispersed in the literature. China is a developing country and has been rapidly moving towards industrialisation from the 1980s resulting in changes in environment and life style. Studies about the incidence and the trends of ectopic pregnancy, miscarriage and hydatidiform mole in China are limited. Therefore, in order to analyse the trends and ages of these major pathological pregnancies in China, and the association among three major pathological pregnancies, we performed a retrospective analysis of data obtained from the largest and top-ranked obstetrics and gynaecology specialised university teaching hospital serving diverse urban and rural areas over an 11 years period in China.

## Methods

This study was approved by the Ethics Committee of The Hospital of Obstetrics & Gynaecology, Fudan University, China (issued on13th October 2014, reference number 201,443).

Data on this retrospective study was collected from an electronic database in The Hospital of Obstetrics & Gynaecology, Fudan University of China from between 2003 and 2013. The Hospital of Obstetrics & Gynaecology Fudan University is the largest specialised referral obstetrics & gynaecology teaching hospital located in Shanghai which is the largest city in China with population of 20 million and a wealthy region. The Hospital of Obstetrics & Gynaecology, Fudan University serves a diverse urban and rural population with between 1 to 1.3 million outpatients and 30, 000 to 40,000 inpatients annually. Data on all patients with a diagnosis of ectopic pregnancy, hydatidiform mole and miscarriage administered to the hospital were collected from the hospital’s electronic database according to the medical records of patients including clinical, intra-operation and pathological findings. Data included the numbers of newly diagnosed ectopic pregnancy, hydatidiform mole and miscarriage and the age of patients at diagnosis. Patients who miscarried due to ectopic pregnancy or hydatidiform mole were not counted towards the total number of miscarriage.

Ectopic pregnancy, hydatidiform mole and miscarriage are referred to as pathological pregnancies in this study.

All the women in this study were Han ethnicity.

### Statistical analysis

To analysis the trend of number of ectopic pregnancies, miscarriage and molar pregnancies over time, linear regression on the number of pregnancies with year as explanatory variable was used. To analyse age trend in molar pregnancy patients, linear regression on log transformed age with year as explanatory variable was used. Age was log transformed due to skewness. The data were analysed using SAS software, version 9.4 (SAS Institute Inc., Cary, NC, USA) with *p* <0.05 being considered as statistically significant.

## Results

### The absolute numbers of ectopic pregnancy, hydatidiform mole and miscarriage are increased from 2003 to 2013

The total case number of women with pathological pregnancies included in this study was 22,511 during the study period of 11 years. Of them, the total number of ectopic pregnancy, hydatidiform mole and miscarriage admitted to our hospital that are included in this study was 14,528 (64.5 %), 1086 (4.9 %), 6897 (30.6 %) respectively. The total case number of women with pathological pregnancies significantly increased by 3.5 folds from 933 cases in 2003 to 3233 cases in 2013 over the study period.

**Fig. 1:**
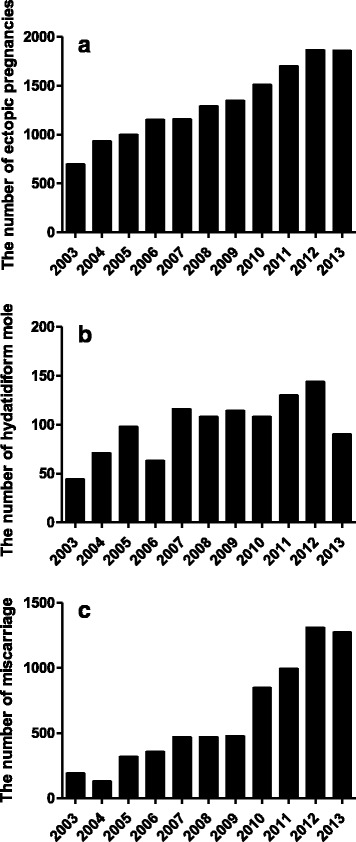
The significant increase in the number of ectopic pregnancy (**a**), hydatidiform mole (**b**) and miscarriage (**c**) from 2003 to 2013 in China

The case number of women with newly diagnosed ectopic pregnancy significantly increased annually, from 698 cases in 2003 to 1860 cases in 2013 (Fig. 1a, *p* ≤ 0.0001). The case number of women with newly diagnosed hydatidiform mole significantly increased annually, from 44 cases in 2003 to 90 cases in 2013 (Fig. 1b, *p* = 0.01). The case number of women with newly diagnosed miscarriage significantly increased annually, from 192 cases in 2003 to 1274 cases in 2013 (Fig. 1c, *p* ≤ 0.0001).

In all these pathological pregnancies, the proportion of ectopic pregnancy was significantly reduced from 74.8 % (698 cases) in 2003 to 57.6 % (1860 cases) in 2013 (*p* < 0.0001, Chi-square test). The proportion of hydatidiform mole also reduced significantly from 4.7 % in 2003 and 2.8 % in 2013 (*p* = 0.003). However, the proportion of miscarriage significantly increased from 20.5 % in 2003 to 39.6 % in 2013 (*p* < 0.0001).

### Age of women with ectopic pregnancy, hydatidiform mole and miscarriage at diagnosis from 2003 to 2013

The median age of women with ectopic pregnancy or hydatidiform mole or miscarriage was 29 years (range 16–58years), 28 years (range 16–57years) and 29 years (range 17–56years) respectively over the study period of 11 years. The median age of women at diagnosis with hydatidiform mole was significantly increased from 25.5 years in 2003 to 29.5 years in 2013 (Table 1). Linear regression on log transformed age showed the increase in age was statistically significant (*p* = 0.002). However, the median age of women at diagnosis with ectopic pregnancies or miscarriage was not changed between 2003 and 2013 (Table 1).

**Table 1:** Trends of age at diagnosis of women with ectopic pregnancy, hydatidiform mole and miscarriage between 2003 and 2013

	Ectopic pregnancy (median, range)	Hydatidiform mole (median, range)	Miscarriage (median, range)
2003	29 (16–51)	25.5 (17–53)	28 (18–54)
2004	28 (17–54)	26 (19–50)	29 (20–46)
2005	28.5 (17–50)	26.5 (18–49)	28 (19–46)
2006	28 (17–53)	28 (18–53)	28 (18–55)
2007	29 (17–52)	27.5 (18–53)	28 (17–45)
2008	29 (17–56)	29 (18–54)	28 (17–53)
2009	29 (17–48)	27 (17–52)	28 (17–47)
2010	28 (18–50)	28 (17–51)	28 (17–56)
2011	29 (16–54)	28 (18–57)	29 (17–48)
2012	29 (17–56)	28.5 (16–53)	29 (18–52)
2013	29 (16–46)**	29.5 (17–56)*	29 (17–52)**

* *p* = 0.002, linear regression on log transformed age from 2003 to 2013

**P > 0.05, linear regression on log transformed age from 2003 to 2013

### Age-specific distribution of ectopic pregnancy, hydatidiform mole and miscarriage from 2003 to 2013

We then analysed the age specific distribution of women with ectopic pregnancy, hydatidiform mole and miscarriage at diagnosis. Overall, the most frequent age of women with pathological pregnancies was 20–29 years old (ectopic pregnancy, 51.8 % or hydatidiform mole, 55.7 % or miscarriage, 56.1 %), which were significantly higher than other age range (Table 2).

**Table 2:** Overall age-specific distribution in the number of women with ectopic pregnancy, hydatidiform mole and miscarriage

	Ectopic pregnancy (number, percentage)	Hydatidiform mole (number, percentage)	Miscarriage (number, percentage)
Under 20 years	266 (1.8 %)	44 (4 %)	80 (1.1 %)
20–29 years	7529 (51.8 %)*	605 (55.7 %)*	3863 (56.1 %)*
30–39 years	5916 (40.7 %)	238 (21.9 %)	2600 (37.7 %)
Over 40 years	817 (5.62 %)	199 (18.4 %)	354 (5.1 %)

*p < 0.0001 compared with other age groups

We further analysed the trends of age distribution in women with ectopic pregnancy, hydatidiform mole and miscarriage during the study period of 11 years. As shown in Table 3, the age distribution of women with ectopic pregnancy and miscarriage was not different between 2003 and 2013. While the most frequent age of diagnosis for hydatidiform mole was 21–29 years in both 2003 and 2013, the proportion of total diagnoses at 21–29 years was reduced in 2013 (45.5 %) compared to 2003 (61.4 %). In addition, the proportion of total hydatidiform moles diagnosed in patients over 40 years old was greater in 2013 (30 %) compared to 2003 (15.9 %).

**Table 3:** Age specific distribution of ectopic pregnancy, hydatidiform mole and miscarriage between 2003 and 2013

	Ectopic pregnancy		Hydatidiform mole		Miscarriage	
Age (number, %)	2003	2013	2003	2013	2003	2013
≤20 years old	10 (1.4 %)	17 (0.9 %)*	2 (4.5 %)	4 (4.4 %)*	4 (2.1 %)	17 (1.3 %)*
21–29 years old	353 (50.6 %)	918 (51.8 %)*	27 (61.4 %)	41 (45.5 %)*	109 (57.1 %)	656 (56.0 %)*
30–39 years old	274 (39.2 %)	817 (40.7 %)*	8 (18.2 %)	18 (20.0 %)*	63 (32.9 %)	533 (37.7 %)*
≥40 years old	61 (8.7 %)	108 (5.8 %)*	7 (15.9 %)	27 (30.0 %)*	15 (7.8 %)	71 (5.1 %)*
total	698	1860	44	90	191	1277

*p > 0.05: the age specific distribution in 2013 compared to that in 2003

### The maternal age increased from 2003 to 2013

**Fig. 2:**
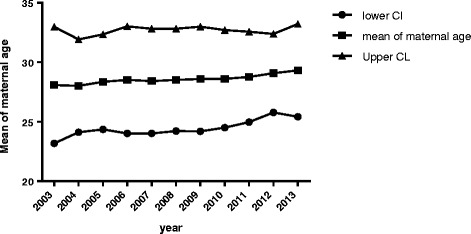
The significant increase in mean of maternal age from 2003 to 2013 in China

The total live birth was 84,223 in this largest obstetrics and gynaecology specialised university teaching hospital during the period of study of 11 years, and the overall mean maternal age for live birth was 28.7 years old. The maternal age for live birth was significantly increased, from 28.1 years old in 2003 to 29.4 years old in 2013 (Fig. 2, *p* < 0.05, ANOVA).

## Discussion

Ectopic pregnancy, hydatidiform mole and miscarriage affect 1–2, 0.1, 10–20 % of pregnancies worldwide, and are the most common types of pathological pregnancies. Geographical regions and ethnicity may vary the incidence of these pathological pregnancies. The trend of incidence of ectopic pregnancy was not changed between 2002 and 2007 in the United States [8], but Asians have been reported to have lower incidence of ectopic pregnancy than Caucasians [14]. The incidence of ectopic pregnancy in China is estimated to be 2.5 % (US Census Bureau, International Data Base, 2004), which is higher than American-Asian (1.19 %) [14]. Asian women are at higher risk of developing hydatidiform mole. The incidence of hydatidiform mole in Southeast Asia is approximate 0.5 % [12], which is significantly higher than that in the United States and the United Kingdom. Interestingly, Caucasians have higher incidence of hydatidiform mole than African Americans. In addition, African Americans are at a higher risk of miscarriage than Caucasian [15].

A number of studies have shown that the incidence of pathological pregnancies is increased in the last decades worldwide [9, 13, 16]. In our current study, data from the largest obstetrics & gynaecology university teaching hospital in China, we found that the absolute case number of women with pathological pregnancies was 3.5fold higher in 2013 than in 2003. Of them, the most common pathological pregnancy is ectopic pregnancy (64.5 %), followed by miscarriage (30.6 %) and hydatidiform mole (4.9 %). Newly diagnosed cases of ectopic pregnancy, hydatidiform mole and miscarriage admitted to our hospital were significantly increased from 2003 to 2013 (2.5 folds increase in ectopic pregnancies, 2 folds increase in hydatidiform mole and 6.5 folds increase in miscarriage). However, the proportion of women with ectopic pregnancy and hydatidiform mole was significantly reduced and the proportion of women with miscarriage was significantly increased in 2013 in all these pathological pregnancies in this study. Taken together, our data suggest that ectopic pregnancy is the leading pathological pregnancy and miscarriage is increasing at a greater rate among the pathological pregnancies at early stages of pregnancy in China. In this study we are not able to estimate the incidence of ectopic pregnancy, hydatidiform mole and miscarriage. This is because our hospital is the largest and top tanked referral hospital (level 3) in Shanghai and in China, a large number of gynaecology patients including patients in this study are referred to our hospital from other hospitals, so their pregnancies were not registered in this hospital.

Although the reasons for the increased number of pathological pregnancies are unclear in China, increased maternal age may contribute to the increase in the numbers of pathological pregnancies. It is well documented that maternal age is associated with an increased risk for miscarriage [17], ectopic pregnancy [4, 18] and hydatidiform mole [19]. The risk for developing hydatidiform mole increases with age [13]. Women under 20 or over 40 years of age have been reported to be at a higher risk of developing ectopic pregnancy, hydatidiform mole and miscarriage compared with women at the ages of 20 to 40 year [19–21]. In our current study, although the majority of patients are 20–29 years old, our data show that the numbers of women with hydatidiform mole who were over 40 years were increased from 7 cases (15.5 %) in 2003 to 27 cases (30 %) in 2013 (Table 3). The maternal age at first pregnancy is increasing in Shanghai, China. Our data from this largest obstetrics & gynaecology hospital in the largest city in China shows that the mean maternal age is increased from 28.1 in 2003 to 29.4 years in 2013. This delayed 16 months of maternal age may be one of the reasons for the increased the numbers of women with ectopic pregnancy or hydatidiform mole or miscarriage in China.

Study suggested that use of an intrauterine device was associated with an increased risk of ectopic pregnancy [4]. Based on Chinese cultural practices, the most common method for contraception is using intrauterine device rather than the use of condoms or oral contraceptive pill. This may result in an increase of ectopic pregnancy. Due to the length of the study and the number of cases included, it was difficult to collect the duration of intrauterine device use and what type of intrauterine device was used.

Another speculation is that environmental factors directly or indirectly influence the reproductive process. A rise in industrialisation and the consequent environmental pollution adversely affects reproductive health [22]. China is rapidly moving towards being an industrialised country since the 1980s and the associated changes in the environment and lifestyle may result in air and water pollution. Recently the particulate matter (PM_2.5_) levels have increased rapidly in the most cites of China, including Shanghai. A number of studies suggested that exposure of air pollution in prenatal time causes adverse birth outcomes, such as low birth weight and preterm birth [23, 24] and spontaneous abortion including early undetectable abortion (reviewed in [22]) as well as abnormal eggs (reviewed in [22]). Hydatidiform mole is caused by an abnormal egg or sperm during fertilisation. Other studies showed that the incidence of ectopic pregnancy was increased 4 times between 1970 and 1987 in the United States due to the result of exposure environmental pollution (reviewed in [22]).

It is important to note that the observations from this study have some limitation. Data was obtained from the largest Obstetrics & Gynaecology university teaching hospital located in Shanghai, China over a period of 11 years. This limited number of women included in the study and restricted geographical areas covered. Although this is not a population based study, data in this study was collected from the largest and top ranked women’s hospital in China. The findings presented in this study may not be representative of China as a whole, but they could provide some useful information in the trends of these common pathological pregnancies. Due to the length of the study and the number of cases included, it was difficult to collect the classification of the each of the diagnosed cases, and the analysis of the subtypes of ectopic pregnancy, hydatidiform mole and miscarriage at presentation could not be conducted.

## Conclusions

In conclusion, our study found that the numbers of the three common pathological pregnancies, ectopic pregnancy, hydatidiform mole and miscarriage were significantly increased since 2003 in China. Ectopic pregnancy is the leading pathological pregnancy and miscarriage is increasing at a great rate among the pathological pregnancies at early stage of pregnancy in China. Although the most frequent age of these pathological pregnancies are not changed during the study period, the trend of age of women with hydatidiform mole and miscarriage appears to shift to over 30 years old. We therefore speculate that the increased maternal age and/or environmental changes may contribute to this change. With continuing trend for delaying the maternal age, and approximately half of all women with ectopic pregnancy, molar pregnancy and miscarriage at diagnosis do not have any known risk factor, therefore early prenatal care is highly important.
